# When Is the Optimal Timing of Surgical Intervention for Severe Functional Tricuspid Regurgitation?

**DOI:** 10.1155/2017/9232658

**Published:** 2017-06-19

**Authors:** Nobuhiro Nakanishi, Masanobu Ishii, Koichi Kaikita, Ken Okamoto, Yasuhiro Izumiya, Eiichiro Yamamoto, Seiji Takashio, Seiji Hokimoto, Toshihiro Fukui, Kenichi Tsujita

**Affiliations:** ^1^Department of Cardiovascular Medicine, Graduate School of Medical Sciences, Kumamoto University, Kumamoto, Japan; ^2^Department of Cardiovascular Surgery, Graduate School of Medical Sciences, Kumamoto University, Kumamoto, Japan

## Abstract

Functional tricuspid regurgitation (TR) is a serious pathology to be noted for severe right heart failure (HF) and poor prognosis; however, the conventional assessment of TR has some limitations and the optimal timing of surgical intervention remains unclear. A 79-year-old Japanese female was admitted to our hospital to undergo cardiac surgery, because edema gradually got worse despite the increase in diuretics. She had a history of atrial fibrillation (AF) and chronic HF due to severe TR and had been treated with a furosemide for leg edema 4 years ago. A transthoracic echocardiogram (TTE), transesophageal echocardiogram, cardiac magnetic resonance imaging, and cardiac pool scintigraphy demonstrated severe functional TR with tricuspid annular dilation, insufficient tricuspid valve coaptation, and reduced right ventricular ejection fraction (EF) but preserved left ventricular EF. In addition, Swan-Ganz catheter study showed normal pulmonary arterial wedge pressure and mean pulmonary arterial pressure. Tricuspid ring annuloplasty was performed with MC3 ring. Postoperative TTE showed trivial TR, and she had no edema with normal sinus rhythm two months later. Annuloplasty to severe functional TR caused by tricuspid annular dilation due to AF dramatically improved right HF. Cardiologist should pay strict attention to the optimal timing of surgical intervention for TR.

## 1. Background

Morbidity of atrial fibrillation (AF) increases annually over the world [1]. In the management of AF, although thromboembolism is a critical problem in clinical practice, functional tricuspid regurgitation (TR) which is caused by tricuspid annular dilation due to AF is also a serious complication leading to severe right heart failure (HF) because previous study showed that increasing TR severity was associated with poor prognosis, independent of biventricular systolic function and pulmonary artery pressure [2]. Surgical intervention which current guidelines recommended may improve the prognosis of patients with severe TR and right HF; however, the conventional assessment of the etiology and severity of TR has some limitations and the appropriate timing of surgery for functional TR without left-sided surgery remains unclear. Recently, transcatheter therapies for treating TR have been developed and could provide better treatment option for isolated severe TR [3–5]. Therefore the optimal timing of surgical intervention including transcatheter therapy needs to be determined urgently.

## 2. Case Presentation

**Figure 1:**
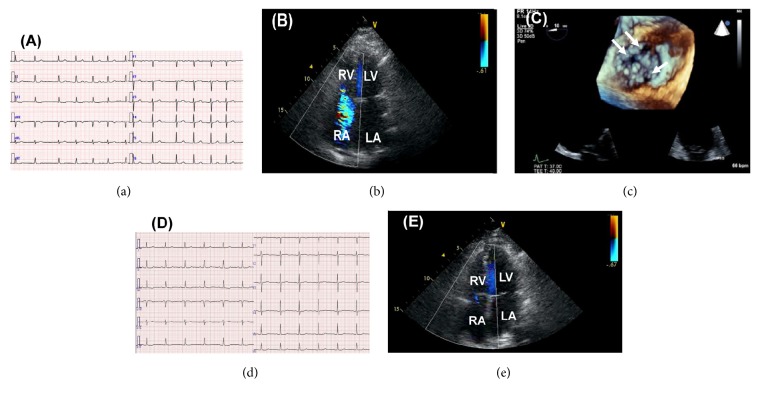
Twelve-lead electrocardiogram (ECG) demonstrating AF rhythm on admission (a) but demonstrating normal sinus rhythm two months after annuloplasty (d). Doppler echocardiography showing severe TR due to insufficient leaflet coaptation on admission (b) but showing the improvement of TR after annuloplasty (e) in the apical view tract at systole. Three-dimensional transesophageal echocardiography showing coaptation defect (arrows) (c). RV: right ventricular; LV: left ventricular; RA: right atrium; LA: left atrium.

**Figure 2:**
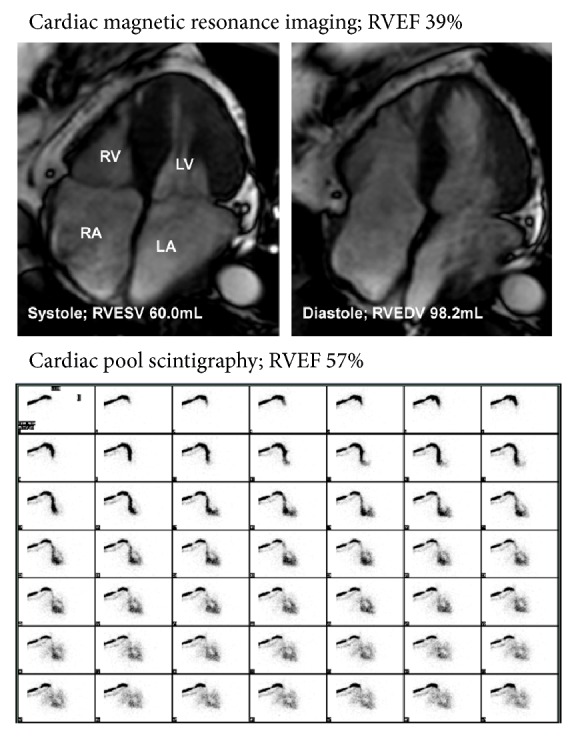
Cardiac magnetic resonance imaging and cardiac pool scintigraphy demonstrating reduced right ventricular ejection fraction (39% and 57%, resp.). RV: right ventricular; LV: left ventricular; RA: right atrium; LA: left atrium; ESV: end-systolic volume; EDV: end-diastolic volume; RVEF: right ventricular ejection fraction.

A 79-year-old Japanese female with a history of AF, hypertension, chronic kidney disease [estimated glomerular filtration rate (eGFR), 53.0 mL/min/1.73 m^2^], and deep venous thrombosis suffering from leg edema, previously diagnosed as right HF due to severe TR free of left-sided heart disease and treated with a diuretic agent furosemide 20 mg/day since 4 years ago, was referred to our institution to undergo cardiac surgery, because her renal function had gradually worsened (eGFR, 30.7 mL/min/1.73 m^2^) with increasing diuretics up to furosemide 40 mg/day for leg edema. On admission, jugular venous distention, extreme pretibial edema, and systolic regurgitant murmur in the third intercostal space at the left sternal border were observed during physical examination. Cardiac biomarkers were elevated [brain natriuretic peptide (BNP), 129.1 pg/mL; high sensitivity troponin T, 0.0206 ng/mL] without liver function abnormality (alanine aminotransferase, 8 U/L; aspartate aminotransferase, 23 U/L; total bilirubin, 0.8 mg/dL) and anemia in the laboratory test.  Table 1 shows other preoperative laboratory data. Electrocardiogram showed AF rhythm (Figure 1(a)). Two-dimensional transthoracic echocardiogram (TTE) showed a tricuspid annular dilation (41 mm), insufficient tricuspid valve coaptation, reduced tricuspid annular plane systolic excursion (16 mm), dilated right atrium, dilated right ventricle, and cardiac output 2.4 L/min (cardiac index, 1.61 L/min/m^2^) with preserved left ventricular ejection fraction (EF) and severe TR was detected without left-sided heart disease by Doppler echocardiography (Figure 1(b)). Three-dimensional transesophageal echocardiography revealed tricuspid annular dilation (40.6 mm) and cusp separation without apparent leaflet tethering (Figure 1(c)). In addition, right ventricular EF was established by cardiac magnetic resonance imaging and cardiac pool scintigraphy (39% and 57%, resp.) (Figure 2). However, there were no abnormalities in the structure of subvalvular and leaflets. Swan-Ganz catheter study revealed that pulmonary arterial wedge pressure was 5 mmHg, mean pulmonary arterial pressure was 8 mmHg, mean right atrial pressure was 4 mmHg, pulmonary vascular resistance index was 155 dynes *∗* sec/cm^5^/m^2^, and cardiac index was 1.46 L/min/m^2^ measured by Fick method. EuroSCORE II was calculated as 2.76%, indicating low-to-moderate perioperative risk. On the basis of these findings and current guideline, tricuspid ring annuloplasty was planned. As the right atrium and ventricle were dilated, the tricuspid valve annulus was also dilated; however, tricuspid valve leaflets were intact. Tricuspid ring annuloplasty with MC3 ring (28 mm) was performed successfully. After the annuloplasty, her edema gradually disappeared with normal sinus rhythm (Figure 1(d)), and postoperative TTE showed trivial TR (Figure 1(e)) and increased cardiac output, 2.8 L/min (cardiac index, 1.88 L/min/m^2^). The plasma BNP was significantly decreased to 41.0 pg/mL at 9 months after surgery. Other postoperative laboratory data are listed in Table 1.

## 3. Discussion

Tricuspid valve disorders had traditionally tended to be ignored compared to left-sided valvular heart disease in spite of the association between increasing TR severity and long-term poor prognosis [2]. The major causes of TR (more than 80%) are functional (secondary) TR with right ventricular and tricuspid annular dilation due to pressure or volume overload, left-sided heart disease, or trauma, whereas primary TR is rare [6]. As in the present case, one of the causes of functional TR is AF, which often develops adverse right ventricular remodeling by altering right ventricular compliance, resulting tricuspid annular dilation, and leaflet coaptation defect [7]. In addition, previous study showed that AF caused more dilation of the tricuspid annulus than the mitral because the annular fibrous skeleton was less developed in the tricuspid than the mitral [8].

Pharmacological therapy using diuretics is effective for systemic congestion in the early phases of the disease; however, such therapy does not affect prognosis. For the improvement of survival, surgical intervention should be performed with the optimal timing. It has been shown that right ventricular dysfunction is important for decision-making in surgery because perioperative adverse right ventricular function was a risk factor for poor prognosis. Kim et al. reported that right ventricular end-systolic area emerged as an independent determinant of clinical outcome after surgery in patients with isolated TR [9]. However, most patients with isolated TR in the previous study [9] had a history of left-sided valve surgery, and no study reported the prognostic impact of perioperative right ventricular function in patients with TR free of left-sided heart disease. In addition, because of complex geometry of right ventricle, right ventricular function cannot be evaluated reliably and reproducibly. In fact, the two imaging modalities (cardiac magnetic resonance imaging and cardiac pool scintigraphy) used in the present case showed quite different right ventricular EF. Therefore, further studies are warranted to clarify the prognostic impact of perioperative right ventricular function using multiple imaging modalities in patients with TR free of left-sided heart disease. Besides right ventricular function, hemoglobin level, TR jet area, and pulmonary artery systolic pressure were also predictors of mortality after surgery in the previous studies [9, 10]. The evaluation of these preoperative risk factors could be needed for improvement of clinical outcome after TR surgery.

Current guideline regarding the indication for TR surgery indicated that TR surgery was recommended for patients with severe functional TR at the time of left-sided valve surgery [11]. However, the optimal timing of TR surgery for patients with isolated functional TR free of left-sided valve heart disease has not been established, as described above. In addition, if patients with severe functional TR were treated with diuretics, TR often would become mild. This transient improvement of TR grade might mislead the optimal timing for TR surgery. Although the timing when diuretics become unresponsive may be the appropriate timing when the TR surgery should be performed, more accurate indicators are needed to determine the optimal timing of surgical intervention for severe TR. In this regard, Dreyfus et al. proposed a new method for assessing functional TR using 3 parameters: TR severity, annular dilation, and mode of leaflet coaptation (extent of tethering) [7]. Based on this staging system, the present case, presenting severe TR, a tricuspid annular dilation (41 mm), and tricuspid leaflet cusp separation without tethering, was staged as category 3, indicating that the appropriate timing of surgery could improve her prognosis and quality of life free of heart failure despite the fact that conventional assessment showed right ventricular dysfunction without pulmonary artery hypertension and other organ failures except for renal dysfunction.

## 4. Conclusions

Annuloplasty to severe functional TR caused by tricuspid annular dilation due to AF dramatically improved right HF. Recently, transcatheter therapies for tricuspid valve disorders are available. Cardiologists should revise their recognition for the assessment and optimal timing of surgical intervention for TR and need to stay aware of the indication of TR surgery even if TR is mild.

## Figures and Tables

**Table 1:** Pre- and postoperative laboratory data.

	Preoperative	Postoperative (9 months after surgery)
Alb, g/dL	3.5	4.3
AST, U/L	8	31
ALT, U/L	23	25
Total bilirubin, mg/dL	0.8	0.8
ChE, U/L	279	Not done
BUN, mg/dL	21	23.5
Creatinine, mg/dL	1.21	1.30
eGFR, mL/min/1.73 m^2^	30.7	27.0
PT-INR	2.3	1.89
APTT, sec	42.4	Not done
BNP, pg/mL	129.1	41.0

Alb: albumin; AST: aspartate aminotransferase; ALT: alanine aminotransferase; ChE: cholesterol ester; BUN: blood urea nitrogen; eGFR: estimated glomerular filtration rate; PT-INR: international normalized ratio of prothrombin time; APTT: activated partial thromboplastin time; BNP: brain natriuretic peptide.

## Abbreviations

TR: Tricuspid valve regurgitation
HF: Heart failure
AF: Atrial fibrillation
eGFR: Estimated glomerular filtration rate
TTE: Transthoracic echocardiogram
EF: Ejection fraction
BNP: Brain natriuretic peptide.
